# Three stage axillary lymphatic massage optimizes sentinel lymph node localisation using blue dye

**DOI:** 10.1186/1477-7800-4-30

**Published:** 2007-12-22

**Authors:** Robert M Kirby, Abdul Basit, Quang T Nguyen, Anthony Jaipersad, Rebecca Billingham

**Affiliations:** 1 Breast Unit, University Hospital of North Staffordshire, Keele University, Stoke-on-Trent, UK

## Abstract

**Aims:**

This paper describes a simple technique of axillary and breast massage which improves the successful identification of blue sentinel nodes using patent blue dye alone.

**Methods:**

Patent blue dye was injected in the subdermal part of the retroaroelar area in 167 patients having surgical treatment for invasive breast cancer. Three stage axillary lymphatic massage was performed prior to making the axillary incision for sentinel lymph node biopsy. All patients had completion axillary sampling or clearance.

**Results:**

A blue lymphatic duct leading to lymph nodes of the first drainage was identified in 163 (97%) of the patients. Results are compared with 168 patients who had sentinel lymph node biopsy using blue dye without axillary massage. Allergic reactions were observed in four patients (1.2%).

**Conclusion:**

Three stage axillary lymphatic massage improves the successful identification of a blue sentinel lymph node in breast cancer patients.

## Introduction

Lymphatic mapping using blue dye, radiolabelled colloid or both to identify the sentinel lymph node in breast cancer surgery is becoming the standard of care. In the validation phase of the ALMANAC trial surgeons achieving a localization rate of = 90% using a combined technique of blue dye and radioisotope proceeded to the randomization phase in which the success rate for localization of the sentinel lymph node was 98% [1]. A number of studies (Table 1) have reported a localization rate in the range of 85–98% using the combined technique, [2-4] However Adwani and Giuliano have reported a 90–95% success rate of localization of the sentinel node using the blue dye alone without and with a breast massage respectively [5,6]. Although both radiocolloid and blue dye are used together by most surgeons, and training should be in both techniques, some experienced surgeons use one or the other almost exclusively. [6,7]

**Table 1:** 

**Author**	**Number of Patients**	**Technique**	Number of SLN*	**SLN Identification rate (%)**
Giuliano 1997	107	Blue Dye Only	1.8	93
Veronesi 2003	649	Radiocolloid only	2.7	98.7
Krag 1998	443	Radiocolloid only	2.5	93
ALMANAC Mansel 2006	1031	Combined Blue Dye and Radiocolloid	2.4	98
Hadjiminas 2005	234	Combined Blue Dye and Radiocolloid	1.38	94.5
Kuehn 2004	1124	Combined Blue Dye and Radiocolloid	2	85

* Average number

We report a technique using a three stage axillary and breast massage after subcutaneous injection of 2 ml of patent blue dye alone. This massage technique applies the principle of manual lymphatic drainage and is based on the physiology of lymphatic flow described originally by Winiwarter (1892) and Vodder (1982). It improves the localisation of a blue lymphatic duct in the axilla and almost always leads to successful identification of the blue sentinel lymph node. [8]

## Methods

**Figure 1:**
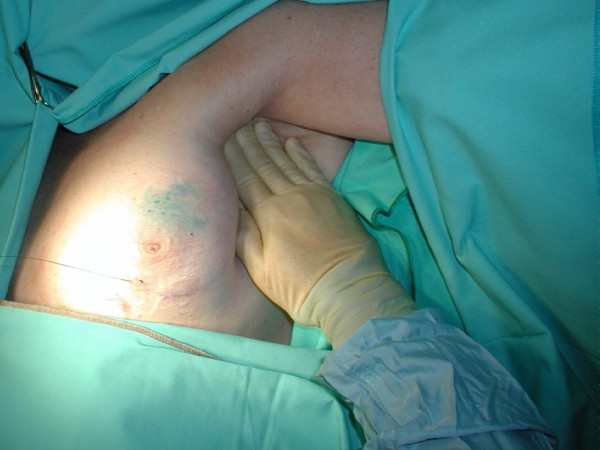
First stage of axillary lymphatic massage.

**Figure 2:**
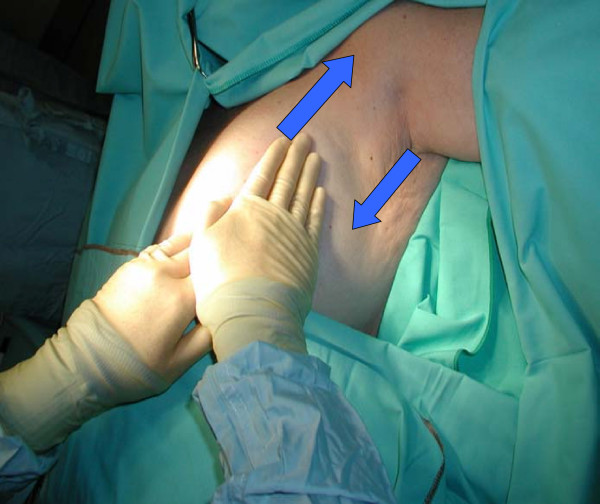
Stage 2 retrograde and stage 3 antegrade massage.

We injected 2 ml of 2.5 per cent undiluted patent blue V sodium dye subdermally in the retroaroelar area at the upper outer quadrant of the breast. The lymphatic massage was performed in three stages. In the first stage the axilla was massaged to empty axillary lymph nodes (Fig. 1). The lymph node massage was performed with the tips of two or three fingers. These were placed over the nodes, and pressure was applied like a gentle "scoop" in the direction of further flow from them. The fingers did not move over the skin; rather, they applied pressure during the "scoop" and released it [8]. In the second stage a retrograde massage was undertaken from the axilla towards the injection site. This was performed using the palm of the hand and fingers using light stroking movements over the skin. The final step was to massage from the injected area towards the axilla (Antegrade massage) to drain the blue dye into lymphatics and axillary lymph nodes (Fig. 2). The three stage massage takes about one minute to complete. It uses light-pressured strokes in a specific direction to stimulate the lymph vessels just below the surface of the skin without increasing blood circulation or reaching the depth of muscle tissue. It was thought necessary to allow about seven minutes before making the axillary incision to provide the blue dye enough time to reach the axillary glands. All patients had completion axillary sampling or clearance.

## Results

In 167 patients with three stage axillary lymphatic massage a blue sentinel lymph node was identified in 163 patients thereby achieving a localisation rate of 97%. We compared the results with our previous technique of simple massage from the injection site towards the axilla (Antegrade massage). A blue sentinel lymph node had been identified in 144 of 168 patients with simple antegrade massage alone, a localisation rate of only 85%.

Four patients (1.2%) developed allergic reaction to patent blue dye which manifested in the form of blisters or wheal and blue hives with bluish discolouration of the skin. One of these patients became hypotensive. All reactions occurred within 45 minutes of the injection and were treated by a single intravenous injection of 200 mg of Prednisolone.

The false negative rate was 6% in the antegrade massage group and zero per cent in the three stage axillary lymphatic massage group. The new technique also improved the sensitivity and specificity from 70 and 88 per cent with simple antegrade massage to 84% and 100% with a three stage massage.

## Discussion

Axillary sentinel lymph node biopsy allows a minimal invasion of the axillary area in order to obtain a limited number of axillary lymph nodes to identify patients who do not have nodal metastases. This avoids a more extensive axillary dissection with its associated complications. It is important that techniques used to localise the axillary sentinel lymph node in breast cancer are optimized to achieve a high identification rate. A combined technique using Tc-99 isotope and patent blue dye has been shown to achieve a localisation rate of up to 98% [1,9,10].

Of the various dyes used for sentinel node localization patent blue dye is currently recommended for sentinel node biopsy in breast cancer patients in the United Kingdom. When injected subdermally in any area of the breast it binds to proteins and is absorbed by the lymphatics and highlights the lymphatic drainage and the sentinel lymph node in the axilla [11]. Three stage lymphatic massage works by first emptying the "reservoir" in the lymph nodes creating an empty space into which lymphatics containing the blue dye can be emptied easily [8].

A localisation rate of over 90% is currently recommended to assure quality in breast cancer surgery [7,12,13]. We report in this study that a localisation rate of 97% is achievable by using blue dye alone with axillary lymphatic massage in three stages. This technique can be useful for breast surgeons working in breast units where facilities for nuclear medicine are not readily available [14]. A blue dye assisted four node sampling can be performed making it a targeted axillary sampling [15]. A comparison of Methylene blue dye and combined dye and radioactive technique has shown that the blue dye technique with breast massage can be as accurate as the combined isotope dye technique [16].
